# Severe E-Cigarette-Induced Esophagitis in a Patient With Eosinophilic Esophagitis: A Case Report

**DOI:** 10.7759/cureus.83563

**Published:** 2025-05-06

**Authors:** Larissa A Lucena, Paulo César da Silva, Verônica S Vale, Sílvio José L Dantas, Rodrigo A Oliveira

**Affiliations:** 1 Health Sciences, Universidade Federal do Rio Grande do Norte, Natal, BRA; 2 Gastroenterology, Casa de Saúde São Lucas, Natal, BRA; 3 Gastroenterology, Gastrocentro, Natal, BRA; 4 Digestive Surgery, Universidade Potiguar, Natal, BRA; 5 Integrated Medicine, Universidade Federal do Rio Grande do Norte, Natal, BRA

**Keywords:** case report, chemical esophagitis, ulcerative esophagitis, upper gastrointestinal endoscopy, vaping

## Abstract

Chemical esophagitis, a rare and potentially life-threatening condition, was diagnosed in a 24-year-old man with food allergies, presumed IgA nephropathy, eosinophilic esophagitis (EoE), and electronic cigarette (e-cigarette) use. After a week of vaping, he developed severe dysphagia, and endoscopy revealed extensive esophageal ulcerations and necrosis. Treatment included total parenteral nutrition, esomeprazole, and sucralfate. Follow-up endoscopy showed diverticular formations and friable lesions. This case underscores the emerging link between vaping and ulcerative esophagitis, especially in patients with pre-existing conditions, highlighting the need for early diagnosis and further research to understand and manage this association.

## Introduction

Electronic cigarettes (e-cigarettes) have emerged as popular alternatives to traditional tobacco, particularly among young adults. Despite their reputation as a safer option, e-cigarettes have been linked to various health concerns, most notably e-cigarette- or vaping-associated lung injury (EVALI) [1]. However, the impact of vaping on the gastrointestinal (GI) tract, especially the esophagus, is poorly characterized and likely underrecognized.

Recent case reports have described esophageal injury associated with vaping, including presentations of severe erosive esophagitis in otherwise healthy individuals [2,3]. The mechanism is not fully understood but may involve direct mucosal irritation, chemical toxicity, and immune-mediated responses triggered by flavoring agents or other components of e-liquids [4,5]. Individuals with pre-existing esophageal disorders, such as eosinophilic esophagitis (EoE), may be particularly vulnerable to these effects.

We report a rare case of extensive esophageal ulceration temporally associated with vaping in a young man with a history of EoE, highlighting a potential link between electronic cigarette use and chemical esophagitis.

## Case presentation

A 24-year-old man presented to the emergency department with acute odynophagia and progressively worsening dysphagia over the past week. Symptoms affected both solids and liquids. He reported the recurrent use of electronic cigarettes, approximately three times per week, using a refillable device with fruit-flavored e-liquids. The patient resided in Brazil but imported the e-cigarette product from Europe. The onset of symptoms occurred shortly after his most recent vaping episode. He denied chest pain, fever, or weight loss.

He had a history of EoE and presumed IgA nephropathy. He also reported known allergies to egg and coconut. He consumed alcohol occasionally in small amounts and denied use of traditional tobacco cigarettes, illicit drugs, or nonsteroidal anti-inflammatory drugs.

At the time of admission, his EoE was clinically inactive. He had been using proton pump inhibitors (PPIs) and sucralfate on an as-needed basis, with no recent exacerbations. Physical examination was unremarkable, and vital signs were within normal limits. Laboratory test results are shown in Table 1.

**Table 1:** Admission laboratory test results. aPTT, activated partial thromboplastin time; PT, prothrombin time; GT, glutamyl transferase; NR, nonreactive; HCV, hepatitis C virus; VDRL, venereal disease research laboratory

Test	Patient’s value	Reference range
Hemoglobin	13.7 g/dL	12.0-15.0 g/dL
Leukocytes	12,610 cells/mm³	4,000-10,000 cells/mm³
Neutrophils	10,466 cells/mm³	2,000-7,000 cells/mm³
Eosinophils	126 cells/mm³	20-500 cells/mm³
Platelet count	235,000 cells/mm³	150,000-400,000 cells/mm³
aPTT	34.3 seconds	28-42 seconds
PT	15.4 seconds	11.0-13.5 seconds
Urea	36 mg/dL	16.6-48.5 mg/dL
Creatinine	1.3 mg/dL	0.70-1.20 mg/dL
Sodium	137 mmol/L	136-145 mmol/L
Potassium	4.1 mmol/L	3.5-5.1 mmol/L
Ionic calcium	1.08 mmol/L	1.16-1.31 mmol/L
Magnesium	2.0 mg/dL	1.7-2.2 mg/dL
Alkaline phosphatase	75 U/L	30-130 U/L
Gamma-GT	49 U/L	5-40 U/L
C-reactive protein	346.8 mg/L	<5.0 mg/L
Urinalysis	Proteins (+), presence of red blood cells, and granular casts	Negative
HIV-1 and HIV-2	NR	-
Hepatitis C (anti-HCV)	NR	-
VDRL	NR	-
Cytomegalovirus, IgG and IgM	NR	-
Herpes simplex I and II, IgG	NR	-

**Figure 1:**
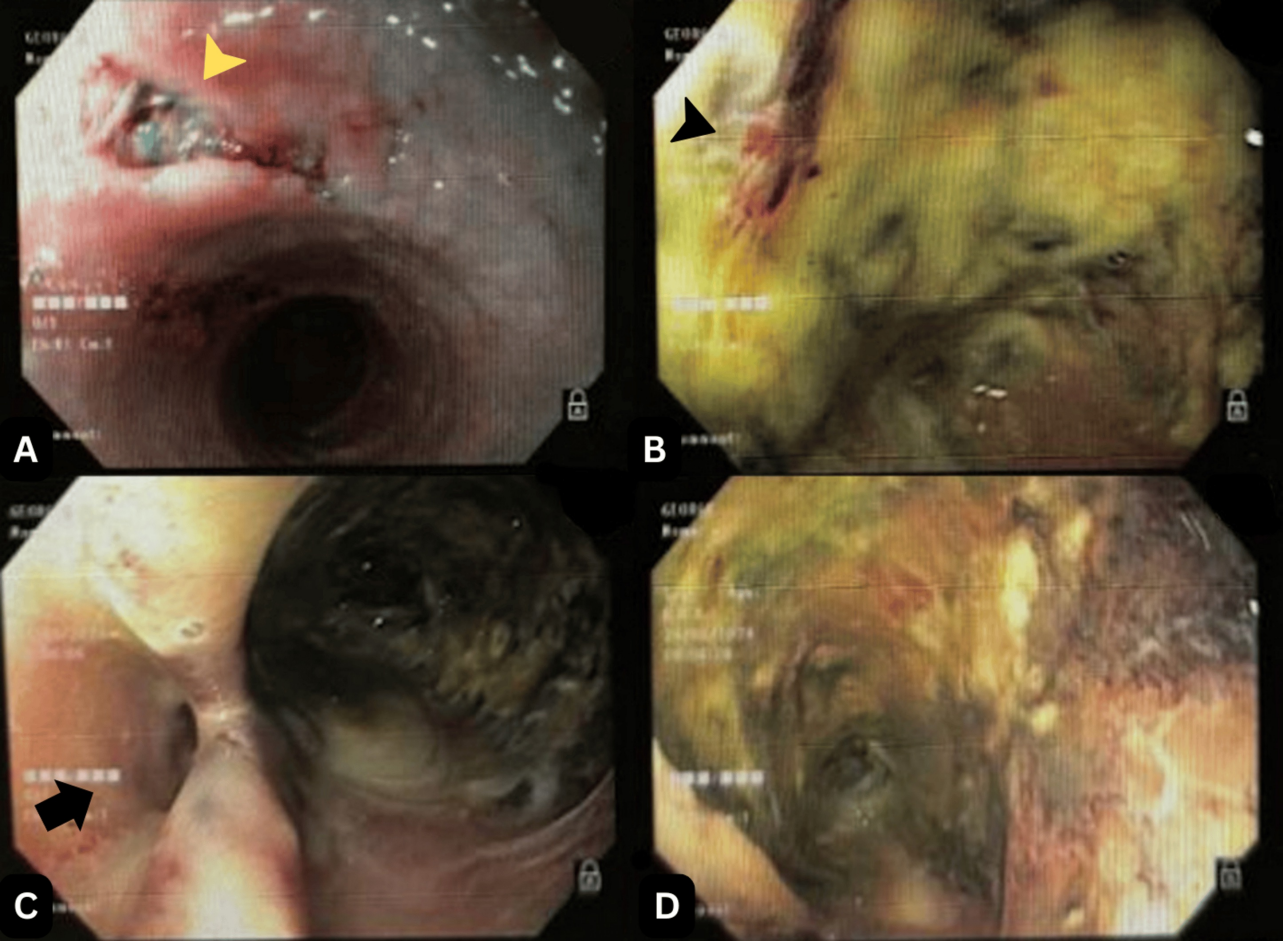
Initial upper gastrointestinal endoscopy revealed extensive ulcerative lesions and necrosis in the esophagus. (A) At 18 cm from the upper dental arcade (UDA), ulcerated, moderately deep lesions are observed (yellow arrowhead). (B) At approximately 25 cm from the UDA, extensive, friable, ulcerated lesions with broad areas of necrosis and thin, delicate mucosa are visualized (black arrowhead). (C and D) A probable fistulous opening is also identified (black arrow).

Upper gastrointestinal endoscopy revealed extensive ulcerative lesions beginning 18 cm from the upper dental arch, which became increasingly friable and circumferential around 25 cm. The mucosa appeared thinned, and a probable fistulous tract was noted (Figure 1). Due to the high risk of perforation, biopsies were deferred. The endoscopic findings were classified as Zargar grade 3A caustic injury.

Thoracic and abdominal computed tomography demonstrated circumferential esophageal wall thickening, distal esophageal pneumatosis, and patchy bilateral pulmonary opacities. A comprehensive infectious workup, including serologies for HIV, hepatitis viruses, herpes simplex virus (HSV), and cytomegalovirus (CMV); blood cultures; and acid-fast bacilli testing, was negative.

The patient was initially managed with total parenteral nutrition for 10 days, followed by a gradual transition to a liquid oral diet. Pharmacological therapy included intravenous esomeprazole and oral sucralfate. He was advised to permanently discontinue vaping.

**Figure 2:**
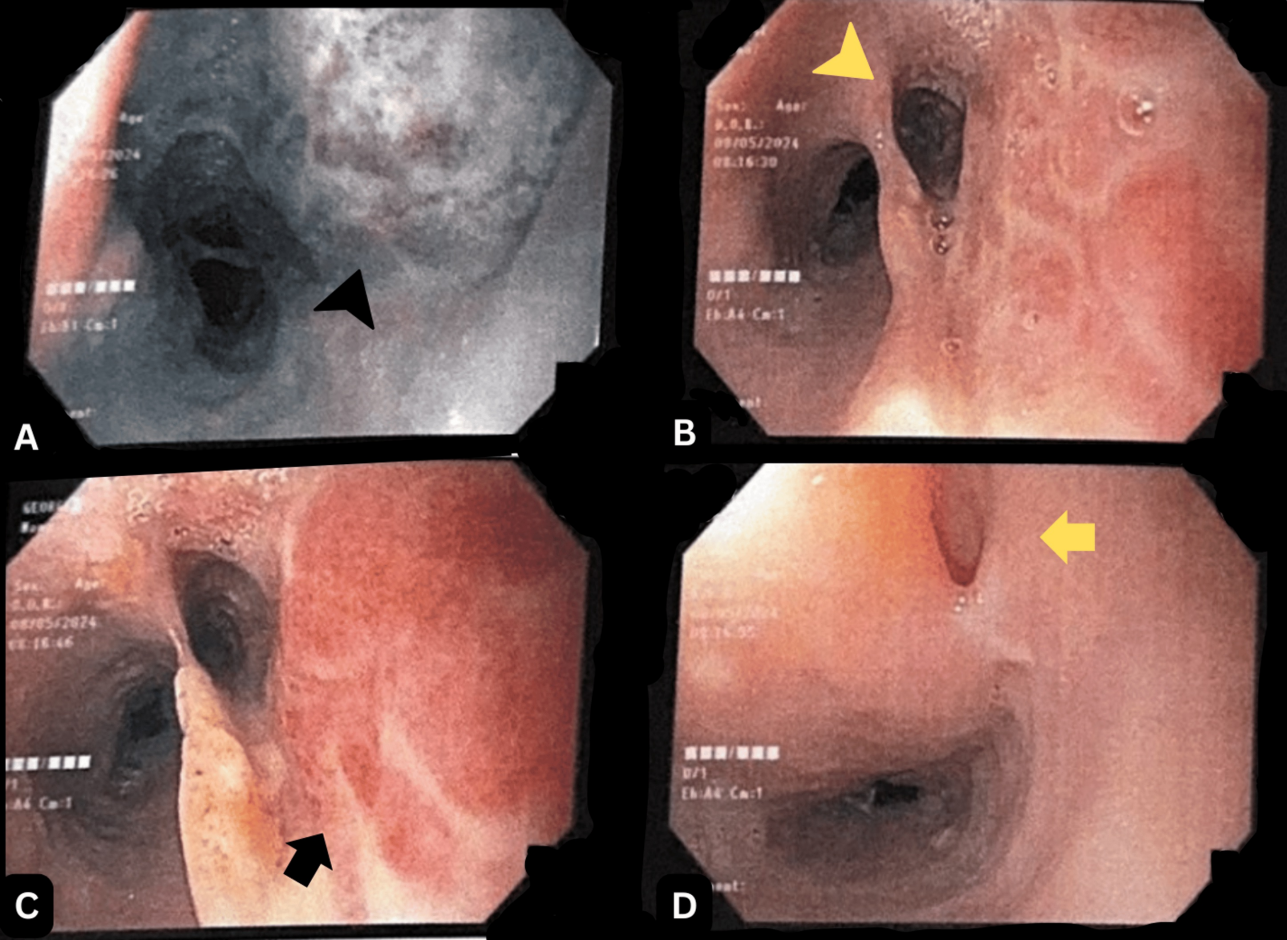
Follow-up endoscopy revealed diverticular formations and friable elevated lesions in the upper and middle esophagus. (A) At 18 cm from the UDA, focal areas of fibrosis are observed (black arrowhead). (B) At approximately 20 cm, a small diverticulum with a mucosal bridge is noted (yellow arrowhead). (C) At around 25 cm from the UDA, on the right lateral wall, an elevated lesion with an erythematous, friable surface upon endoscopic contact is seen (black arrow). (D) Slightly below, at approximately 30 cm from the UDA, another diverticular formation with a mucosal bridge and associated fibrosis is identified (yellow arrow). UDA: upper dental arcade

A follow-up endoscopy performed 30 days later showed marked improvement in mucosal integrity, though residual friable elevated lesions and diverticular formations persisted in the upper and middle thirds of the esophagus (Figure 2). Testing for *Helicobacter pylori* was negative. At that point, due to concern for ongoing mucosal vulnerability and to prevent further complications (as well as an EoE flare), treatment with oral budesonide twice daily and esomeprazole once daily was initiated. The patient reported the complete resolution of dysphagia and was tolerating a regular solid diet at last follow-up.

## Discussion

This case underscores a rare but severe esophageal injury temporally associated with e-cigarette use. While the pulmonary complications of vaping are increasingly recognized, gastrointestinal injuries remain less well-characterized. Our patient, with a history of EoE in remission, presented with extensive esophageal ulcerations and a suspected fistulous tract following recurrent exposure to fruit-flavored vaping products.

The endoscopic findings were classified as Zargar grade 3A, indicating focal necrosis without perforation. The Zargar classification is a widely accepted system for grading caustic esophageal injuries, with higher grades correlating with an increased risk of complications such as strictures and perforation [6].

Although EoE can compromise mucosal integrity, the abrupt onset of symptoms following vaping, combined with negative infectious workup and the severity of endoscopic findings, supports a toxic/chemical etiology rather than an EoE flare. Reviews and similar cases have suggested that heated chemical aerosols in e-cigarette vapor can directly injure the esophageal epithelium [2,3,7,8].

The pathophysiology underlying vaping-induced esophageal injury remains speculative. However, it is hypothesized that the inhalation of heated chemicals and flavoring agents in e-cigarette aerosols may lead to direct mucosal irritation and inflammation. Additionally, nicotine exposure has been shown to alter esophageal pH and motility, potentially exacerbating mucosal damage [2,8-10].

The management of severe esophageal injuries involves supportive care, including nil per os (NPO) status, intravenous PPIs, and mucosal protectants such as sucralfate [10]. In our patient, total parenteral nutrition was initiated due to the extent of mucosal injury. Following stabilization, the patient was transitioned to a liquid diet.

This case emphasizes the need for heightened awareness among clinicians regarding the potential gastrointestinal risks associated with vaping, particularly in individuals with pre-existing esophageal conditions. Further research is warranted to elucidate the mechanisms by which vaping contributes to esophageal injury and to establish evidence-based guidelines for diagnosis and management.

## Conclusions

This case reports a severe Zargar grade 3A caustic esophageal injury likely caused by recurrent e-cigarette use in a patient with controlled EoE. Acute symptoms following vaping, coupled with ulcerative lesions on endoscopy, suggest a chemical etiology. Treatment with parenteral nutrition, esomeprazole, sucralfate, budesonide, and vaping cessation led to symptom resolution. This highlights the gastrointestinal risks of vaping, warranting further research.
